# Optimization of intra-operative electrophysiological localization of the ligament of Marshall

**DOI:** 10.3389/fcvm.2022.1030064

**Published:** 2022-11-03

**Authors:** Sanne J. J. Langmuur, Yannick J. H. J. Taverne, Mathijs S. van Schie, Ad J. J. C. Bogers, Natasja M. S. de Groot

**Affiliations:** ^1^Department of Cardiothoracic Surgery, Erasmus Medical Center, Rotterdam, Netherlands; ^2^Department of Cardiology, Erasmus Medical Center, Rotterdam, Netherlands

**Keywords:** ligament of Marshall, cardiac mapping, cardiac surgery, atrial fibrillation, sinus rhythm, voltage mapping, activation mapping, epicardial mapping

## Abstract

**Background:**

The ligament of Marshall (LOM) may play a role in the pathophysiology of several tachyarrhythmias and accurate electrophysiological localization of this structure is crucial for effective ablation therapy. This study therefore quantifies electrophysiological properties of the LOM, and identifies which electrogram (EGM) recording (uni- or bipolar) and processing technologies [local activation time (LAT) and/or voltage mapping] are most suitable for accurate localization of the LOM.

**Methods:**

The LOM was electrophysiologically identified in 19 patients (mean age 66 ± 14 years; 12 male) undergoing elective cardiac surgery using intra-operative high-density epicardial mapping, to quantify and visualize EGM features during sinus rhythm.

**Results:**

Only a third of LOM potentials that were visualized using unipolar EGMs, were still visible in bipolar activation maps. Unipolar LOM potentials had lower voltages (P50: LOM: 1.51 (0.42–4.29) mV vs. left atrium (LA): 8.34 (1.50–17.91) mV, *p* < 0.001), less steep slopes (P50: LOM: –0.48 (–1.96 to –0.17) V/s vs. LA: –1.24 (–2.59 to –0.21) V/s, *p* < 0.001), and prolonged activation duration (LOM: 20 (7.5–30.5) ms vs. LA: 16.5 (6–28) ms, *p* = 0.008) compared to LA potentials. Likewise, bipolar LOM voltages were also smaller (P50: LOM: 1.54 (0.48–3.28) mV vs. LA: 3.12 (0.50–7.19) mV, *p* < 0.001).

**Conclusion:**

The LOM was most accurately localized in activation and voltage maps by using unipolar EGMs with annotation of primary deflections in case of single potentials and secondary deflections in case of double or fractionated potentials.

## Introduction

In 1850, Marshall first described a vestigial fold of the pericardium at the back of the left atrium (LA) between the left auricle and the left pulmonary veins (PVs) (1). This is now known as the ligament of Marshall (LOM), which results from embryonic obliteration of the left anterior cardinal vein when the venous system transfers from a symmetric to a right-sided one (2). The LOM contains the vein of Marshall (VOM)—which is also referred to as the oblique vein of the LA—, small sympathetic and parasympathetic nervous fibers, and multiple myocardial tracts toward the LA free wall, known as Marshall bundles (1, 3–10). As a consequence, the LOM is much more than just an embryological remnant: it forms an electro-anatomical connection between the coronary sinus (CS), the left lateral ridge, and the PVs.

The LOM may play an important role in the pathophysiology of various tachyarrhythmias, including ventricular tachycardias, atrioventricular re-entrant tachycardias, ridge-related perimitral atrial flutters, and atrial fibrillation (AF) (11–18). In patients with AF, the LOM may serve as either a source of triggered ectopic activity (8, 14, 19) or as an arrhythmogenic substrate (17, 18).

Because of its arrhythmogenic properties, the LOM has recently gained interest as a target of anti-arrhythmic therapies. This includes additional endocardial or epicardial ablative lesions on the LOM in adjunct to PV isolation, using a catheter-based, surgical or hybrid approach, and the recently introduced technique of VOM ethanol infusion (13, 20–25).

For these ablation approaches, it is of paramount importance to accurately localize the LOM electrophysiologically. However, it is yet unclear what the most suitable mapping approach for this purpose should be. The objectives of this study are therefore to quantify electrophysiological properties of the LOM using an intra-operative high-density epicardial mapping approach, and to identify which electrogram (EGM) recording (uni- or bipolar) and processing technologies [local activation time (LAT) and/or voltage mapping] are most suitable for accurate electrophysiological localization of the LOM.

## Materials and methods

### Study population

Patients undergoing elective open-heart surgery at the Erasmus Medical Center Rotterdam were eligible for inclusion. Exclusion criteria were hemodynamic instability, usage of inotropic agents, emergency cardiac surgery or redo cardiac surgery. All patients signed informed consent to participate in the study protocol approved by the institutional ethical committee (MEC2010-054/MEC2014-393) (26, 27). Patient characteristics were collected from the electronic medical records. The study was conducted according to the principles of the Declaration of Helsinki.

### Mapping procedure

**FIGURE 1:**
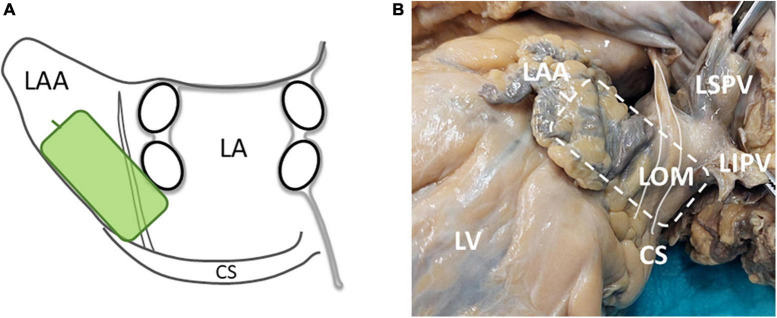
**(A)** A schematic overview of the position and orientation of the electrode array (green rectangle) on the LA. **(B)** A picture of the LOM in a human specimen and the location of the electrode array during LOM mapping (striped rectangle). The white solid line marks the LOM. CS, coronary sinus; LA, left atrium; LAA, left atrial appendage; LIPV, left inferior pulmonary vein; LOM, ligament of Marshall; LSPV, left superior pulmonary vein; LV, left ventricle.

Intra-operative high-density epicardial mapping was performed during sinus rhythm (SR), prior to commencement of extracorporeal circulation, as previously described in detail (26, 27). A custom-made 192-electrode array (interelectrode distance 2 mm, electrode diameter 0.45 mm) was used to record unipolar EGMs for 5 s. This study was part of a more elaborate mapping protocol, that has been described in detail before (27). As this study focused on electrophysiological identification of the LOM, which courses anatomically from the CS obliquely above the LA appendage and lateral to the left PVs, only the LA mapping location was included for analysis (3). At this mapping location, the electrode array was positioned from the lower border of the left inferior PV toward the LA appendage (Figure 1).

A steel wire attached to the thoracic subcutaneous tissue served as the indifferent electrode and a temporal bipolar epicardial pacemaker wire in the right atrial appendage as the reference electrode. Recordings included a surface electrocardiogram (ECG) lead I, a calibration signal of 1,000 ms and 2 mV, a bipolar reference EGM and all unipolar EGMs. After amplification (gain 1,000), filtering (bandwidth 0.5–400 Hz), sampling (1 kHz), and analog to digital conversion (16 bits), all data were stored on a hard disk. Bipolar EGMs were created by subtracting two neighboring unipolar EGMs in horizontal and vertical direction. These bipolar EGMs were subsequently filtered (30–400 Hz).

### Data processing

EGMs were analyzed semi-automatically using custom-made software that annotates the negative slope of each atrial deflection when it was at least–0.05 V/s. Within each potential, the steepest segment of each negative deflection was defined as the LAT; the steepest negative deflection was labeled the primary deflection. Potentials with a single negative deflection (single potentials) only contain a primary deflection. In case of double or fractionated potentials, all others deflections were labeled as secondary deflections.

Premature atrial complexes and activation maps with simultaneous activation were excluded from analysis. Annotations were all manually checked by two investigators.

Color-coded local activation maps were constructed to investigate spatial activation patterns. Conduction delay was defined as minimal difference in LAT between adjacent electrodes of 7–11 ms and conduction block as ≥ 12 ms (28). Potentials were subdivided into single (one deflection), short double (two deflections with a deflection interval < 15 ms), long double (two deflections with a deflection interval ≥ 15 ms), or fractionated (≥ 3 deflections) potentials.

Within bipolar EGMs, potentials were identified using a timeframe of 200 ms surrounding unipolar LATs. Bipolar LAT was defined as the maximum absolute voltage within this timeframe (29). In order to compute the bipolar peak-to-peak voltage of the LA and LOM separately, unipolar activation times of the LA and LOM deflections were used to distinguish between bipolar LA and LOM potentials; the maximum absolute voltage was used as LA and LOM bipolar voltage.

### Identification of ligament of Marshall by different signal processing techniques

Mapping data were screened for activation maps covering the LOM by consensus of two investigators.

For this purpose, two different signal processing techniques were applied to construct LAT maps: (1) annotation of only primary deflections and (2) annotation of both primary and secondary deflections, in case of double or fractionated potentials. In this case, the LAT of the latest deflection was visualized (30). Likewise, two different types of voltage maps were constructed, using either the peak-to-peak amplitude of the primary deflection or the peak-to-peak amplitude of the largest secondary deflection.

As demonstrated in Figure 3, LOM was presumed to be present in an activation map if the following criteria were met. An activation map should contain a circumscriptive area (1) from which double or fractionated potentials are recorded, (2) which is bordered by either two parallel lines of conduction block or one line of conduction block and one line of conduction delay. Patients were excluded from analysis if the full length of this area was either parallel to the border of the electrode or the area was activated simultaneously.

### Data analysis

In patients in whom a LOM was electrophysiologically identified, the first deflection of each double or fractionated potential was classified as LA potential, and all other deflections were classified as LOM potentials. In case of accidental overlap of adjacent mapping locations, whilst both locations included the LOM, the recording in which the largest part of the LOM was visible was included for analysis.

From each unipolar EGM, the peak-to-peak amplitudes and slopes were measured of both LA and LOM potentials. Total duration of LOM activation was defined as the time difference between the first and last LAT of the area containing LOM potentials. Duration of LA activation was calculated as the time difference between the first and last LAT in this same area.

Potential fractionation duration was defined as the time difference (ms) between the first and the last deflection. The maximum time difference between the LOM and the surrounding LA tissue was defined as maximum conduction time (CT_max_).

### Statistical analysis

Data were tested for normality using histograms, QQ-plots and Shapiro-Wilk tests. Continuous variables were reported as mean ± SD if distributed normally and as median (range) otherwise for patient characteristics. Categorical variables were given as number (percentage).

For each individual patient, the 10th, 50th, and 90th percentile of unipolar and bipolar voltages and unipolar slopes, were calculated as a summary measure per patient, separately for the LA and LOM potentials. These were then presented as median with range and compared to see if a difference could be identified between LA and LOM potentials. A similar analysis was performed for the median duration of LOM and LA activation for each patient.

Wilcoxon signed rank tests were performed to compare characteristics of LA and LOM potentials. A *p*-value < 0.05 was considered statistically significant. Data were analyzed using R (version 4.0.3; R Foundation for Statistical Computing, Vienna, Austria).

## Results

### Patient selection

LA mapping locations obtained from 108 patients were screened for the presence of the LOM. A total of 89 patients were excluded because they did not meet the inclusion criteria. Thus, 19 patients were further analyzed.

### Patient characteristics

Table 1 shows characteristics of the 19 patients [mean age: 65.5 ± 13.8 years, male sex: 12 (63.2%)]. Most patients underwent coronary artery bypass graft (CABG) surgery [9 (47.4%)]; others had aortic [4 (21.1%)] or mitral [4 (21.1%)] valve repair or replacement, surgery for congenital heart disease [7 (36.8%)] or arrhythmia surgery [4 (21.1%)]. A history of AF prior to surgery was present in 5 (26.3%) patients, of whom 3 (16.7%) had paroxysmal and 2 (11.1%) persistent AF.

**TABLE 1:** Patient characteristics.

	Overall (*n* = 19)
Age (years)	66 ± 14
Male sex	12 (63.2%)
**Operation indication**	
CABG	9 (47.4%)
AVD	4 (21.1%)
MVD	4 (21.1%)
CHD	7 (36.8%)
Maze	4 (21.1%)
**Preoperative AF**	
None	14 (73.7%)
Paroxysmal	3 (15.8%)
Persistent	2 (10.5%)
BMI	28.5 ± 5.09
Hypertension	11 (57.9%)
Dyslipidemia	6 (31.6%)
Diabetes mellitus	4 (21.1%)
Myocardial infarction	6 (31.6%)

AF, atrial fibrillation; AVD, aortic valve disease; BMI, body mass index; CABG, coronary artery bypass graft; CHD, congenital heart disease; MVD, mitral valve disease. Continuous variables are presented as mean ± SD. Categorical variables are presented as number (percentage).

### Mapping data characteristics

A total of 22,725 potentials were included for analysis, of which 19,536 (86.0%) were classified as LA potentials and 3,189 (14.0%) as LOM potentials (ca. 6 SR beats per patient). This resulted in a median of 1,098 (744–1,316) LA potentials and 165 (28–369) LOM potentials per patient.

### Patterns of activation at the ligament of Marshall area

**FIGURE 2:**
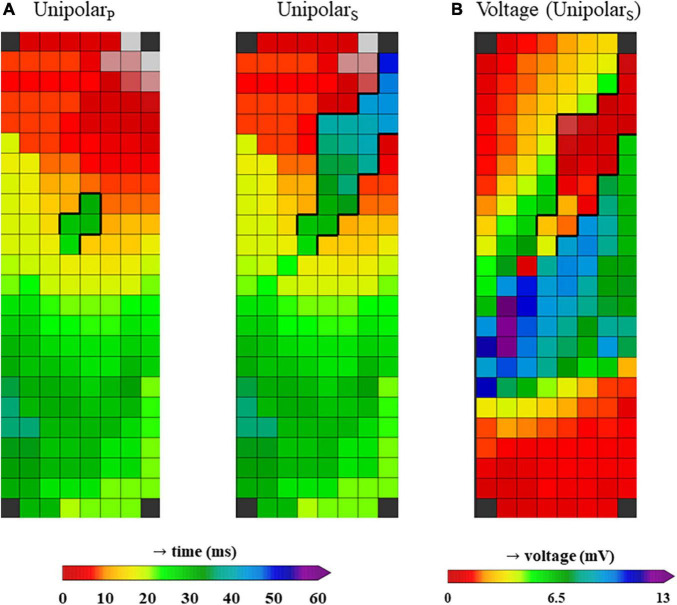
**(A)** Two activation maps from the same patient, constructed using unipolar LATs from only primary deflections (unipolar_P_, left) and a combination of primary deflections in case of single potentials and secondary deflections in case of double or fractionated potentials (unipolar_S_, right). **(B)** Voltage map from the same patient, using the voltages from unipolar primary deflections and the secondary deflections with the largest peak-to-peak amplitude (in case more than one secondary deflection was present).

Figure 2 shows color-coded activation maps obtained from the same beat of a 30-year old female patient who underwent surgery for closure of an atrial septal defect and tricuspid valve repair. This figure illustrates that the LOM can be most easily identified when not only primary unipolar deflections are annotated (unipolar_p_), but also the secondary deflections (unipolar_s_) (Figure 2A). When constructing a voltage map in which either primary deflections, in case of single potentials, or secondary deflections, in case of double and fractionated potentials, are presented, the LOM is easily recognizable as an area containing potentials with lower voltages (Figure 2B).

**FIGURE 3:**
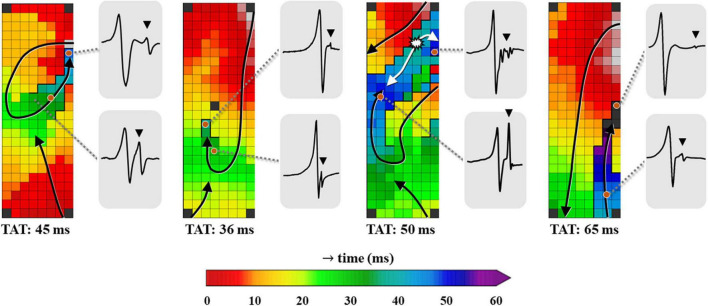
Four examples of color-coded activation maps containing LOMs derived from different patients, constructed using all unipolar deflections. Examples of corresponding EGMs recorded at distinct locations of the LOM are shown outside the activation map, demonstrating the variable morphology of LOM potentials. EGM, electrogram; LOM, ligament of Marshall; TAT, total activation time. The bold black lines represent lines of conduction block and the black triangle in the EGM indicates a LOM potential. The white asterisk indicates a focal pattern of activation.

Using the annotation of secondary deflections to construct activation maps, different patterns of activation were identified. Figure 3 presents four examples of activation maps in which a LOM is visible. These examples show that there is considerable inter-individual variation in activation patterns, size and the total activation time of the LOM.

Median total activation time of the LOM was longer than median total activation time of the surrounding LA tissue [LOM: 20 (7.5–30.5) ms vs. LA: 16.5 (6–28) ms, *p* = 0.008]. The maximum conduction delay between the LOM and the surrounding LA tissue (CT_max_) in each patient ranged between 16 and 65 ms (median: 38 ms).

### Characteristics of unipolar electrograms

Unipolar potentials recorded from the LOM area consisted of short double (34.4%), long double (57.1%), or fractionated potentials (8.5%).

**FIGURE 4:**
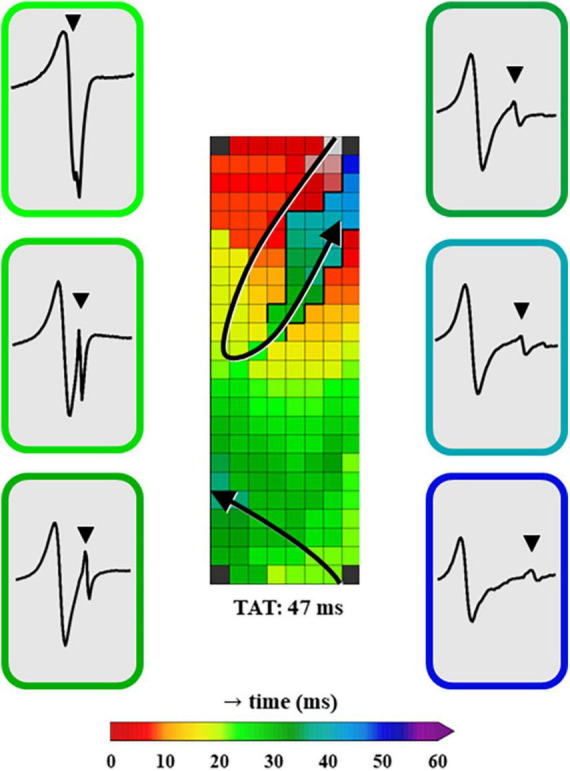
A color-coded activation map, constructed using unipolar EGMs in which all negative deflections are annotated. Additionally, examples of EGMs in the LOM area recorded at distinct moments of LOM activation are shown. The EGMs are depicted in chronological order of LOM activation and the color surrounding each EGM represents the LAT of the recording site within the LOM. EGM, electrogram; LAT, local activation time; LOM, ligament of Marshall; TAT, total activation time. The bold black lines represent lines of conduction block and the black triangle in the EGM indicates a LOM potential.

Figure 4 shows a typical example of a color-coded activation map obtained from the same patient as in Figure 2, annotated using primary deflections of single potentials or secondary deflections of double or fractionated potentials. A few EGMs recorded within the LOM area are illustrated, in which the first deflection clearly represents the LA activation and the other, usually smaller deflections, the LOM activation.

As demonstrated in Table 2, characteristics of unipolar LOM EGMs and remaining LA EGMs differed significantly. Compared to LA potentials, median peak-to-peak amplitudes of LOM potentials were significantly lower (P50: LOM: 1.51 (0.42–4.29) mV vs. LA: 8.34 (1.50–17.91) mV, *p* < 0.001). Also, the median slope of LOM potentials was less steep (P50: LOM: –0.48 (–1.96 to –0.17) V/s vs. LA:-1.24 (–2.59 to –0.21) V/s, *p* < 0.001).

**TABLE 2:** Characteristics of unipolar EGMs.

Unipolar EGMs	Value	Ligament of Marshall	Left atrium	*P*-value
Voltage (mV)	P50	1.51 (0.42–4.29)	8.34 (1.50–17.91)	<0.001
	P10	0.62 (0.19–2.14)	4.37 (0.80–7.71)	<0.001
	P90	3.86 (0.75–24.47)	15.52 (2.65–49.07)	<0.001
Slope (V/s)	P50	–0.48 (–1.96 to –0.17)	–1.24 (–2.59 to –0.21)	<0.001
	P10	–1.40 (–12.24 to –0.35)	–4.14 (–17.96 to –0.46)	<0.001
	P90	–0.16 (–0.87 to –0.06)	–0.40 (–1.01 to –0.09)	<0.001
CTmax (ms)	max	38 (16–65)		
Duration of activation (ms)	P50	20 (7.5–30.5)	16.5 (6–28)	0.008

CT_max_, maximum conduction time between two neighboring electrodes; EGM, electrogram.

Values are presented as median (range).

In a median of 98.4% (71.5–100%) of the unipolar potentials per patient, LOM potentials had lower voltages than LA potentials and 30.4% (0–45.7%) of their slopes were steeper than the LA potentials.

### Characteristics of bipolar electrograms

Bipolar activation maps were created using LATs of bipolar EGMs recorded in both vertical and horizontal direction. As the moment of LAT of the bipolar EGM is represented by the moment of the maximum absolute bipolar voltage, the maximum voltage determines which part of the LOM is visible on the corresponding bipolar activation map (29).

**FIGURE 5:**
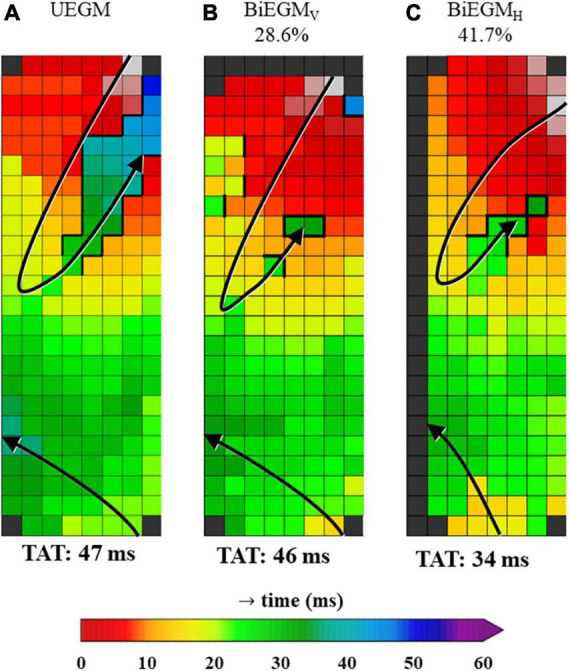
Unipolar and corresponding bipolar vertical and bipolar horizontal activation map obtained from one patient. The percentages on top of the activation maps show how often the amplitude of the LOM was larger than the amplitude of LA tissue for bipolar activation maps. **(A)** Map was constructed using unipolar EGMs in which all negative deflections were annotated (UEGM). **(B)** Map was constructed using bipolar EGMs, created by subtracting EGMs in vertical direction (BiEGM_V_). **(C)** Map was constructed using bipolar EGMs, created by subtracting EGMs in horizontal direction (BiEGM_H_). EGM, electrogram; LA, left atrium; LOM, ligament of Marshall; TAT, total activation time. The bold black lines represent lines of conduction block.

Figure 5 demonstrates an example of a unipolar and corresponding bipolar activation maps, obtained from the same patient as used in Figures 2, 4. In the bipolar activation maps, the mapping area representing LOM activation is smaller, as compared to the unipolar activation map that was constructed using both primary and secondary deflections. The unipolar activation map clearly shows a larger continuous area of LOM activation.

Bipolar voltage characteristics of the LOM and LA potentials recorded from all patients are listed in Table 3. Comparing the reconstructed bipolar EGMs of the LOM and the LA in horizontal and vertical direction, respectively, only 36.7% (0–51.7%) and 30.0% (0–59.5%) of bipolar LOM voltages were larger than bipolar LA voltages. Combining data from both directions, this percentage increased to 35.1% (0.7–54.5%). Thus, only a median of respectively, 36.7, 30.0, or 35.1% of the LOM that is visible in a unipolar activation map in which all negative deflections are annotated, is also visualized in bipolar activation maps.

**TABLE 3:** Characteristics of bipolar EGMs.

Bipolar EGMs	Value	Ligament of Marshall	Left atrium	*P*-value
Horizontal + vertical voltage (mV)	P50	1.54 (0.48–3.28)	3.12 (0.50–7.19)	<0.001
	P10	0.47 (0.22–1.79)	0.87 (0.13–2.72)	0.007
	P90	3.90 (0.94–13.07)	10.99 (1.39–19.78)	<0.001
% LOM > LA—horizontal	P50	36.7% (0–51.7%)		
% LOM > LA—vertical	P50	30.0% (0–59.5%)		
% LOM > LA—all	P50	35.1% (0.7–54.5%)		

EGM, electrogram; LA, left atrium; LOM, ligament of Marshall.

Values are presented as median (range).

Comparable to unipolar EGMs, median voltages of bipolar EGMs were higher in LA than in LOM (P50: LOM: 1.54 (0.48–3.28) mV vs. LA: 3.12 (0.50–7.19) mV, *p* < 0.001).

## Discussion

The ligament of Marshall has gained interest in the field of electrophysiology for its arrhythmogenic properties and therapeutic possibilities related to AF. It is therefore important to accurately localize the LOM electrophysiologically, although the most optimal mapping approach for this purpose has not yet been elucidated.

The key finding of our study is that unipolar EGMs are more useful than bipolar EGMs in localizing the LOM, in particular LAT and voltage mapping of primary deflections of single potentials combined with secondary deflections of double and fractionated potentials. Due to differences in activation direction, the LOM that was identified using unipolar EGMs, remained only partially visible in the bipolar activation maps. Therefore, unipolar EGMs are preferred to accurately localize a LOM.

### Electrogram characteristics

Bipolar LOM potentials were first described by Scherlag et al. (8). As reported previously, LOM potentials consist of two deflections of which the first corresponds to local left atrial activation and the second, narrower and smaller deflection to LOM activation (13, 15, 31). However, this sequence depends on the excitation pattern of the atria, which is variable even during sinus rhythm (32).

The majority of LOM potentials measured in this study consisted of two deflections; these double potentials are generally related to areas of conduction block (33). LOM potentials recorded with either uni- or bipolar technique had smaller voltages and less steep slopes compared to LA potentials, as a result of the smaller bundle structure of the LOM (3). However, as expected, there were considerable differences in bipolar voltage according to the recording direction. In a third of bipolar EGMs, LOM potentials were larger compared to LA potentials. Hence, the LOM was less detectable in bipolar than in unipolar activation maps.

In canine atria, Scherlag et al. measured an interval between LA and LOM bipolar EGMs of 60 ms or more at the most distal point in the LOM (8). In humans, a mean interval of 73 ms measured by a multipolar catheter inside the VOM has been reported (13). The maximum time difference between LOM and LA tissue measured in the current study population was much shorter [38 (16–65) ms]. Smaller time differences may be explained by the fact that our array did not cover the entire VOM and hence the most distal point of the LOM with the largest time difference could have been missed. In our study population, we also measured time differences up to 65 ms. Additionally, activation of the LOM was considerably prolonged compared to the surrounding LA tissue of comparable lengths. This localized slowing of conduction could partly explain the role of the LOM in initiation or perpetuation of atrial tachyarrhythmias.

Prior endo-epicardial mapping studies demonstrated that—except for areas of endo-epicardial asynchrony—features of endo- and opposite epicardial EGMs are comparable (34). Hence, the results of our study are also relevant for endovascular mapping approaches. As expected, all unipolar and bipolar LOM potentials consisted of double or fractionated potentials reflecting local asynchronous activation of underlying superimposed LOM and LA tissue.

### Anatomy and activation patterns

The LOM is the remnant of the embryologic left superior caval vein, but is much more than just a remnant. It has several neuronal and muscular morphological features, comprising the CS musculature, PV sleeves and LA free wall (3, 5, 6). The LOM forms the neural connecting pathway between intrathoracic and intracardiac ganglia with abundant (para) sympathetic to atrial connections that have an interesting topographical neural density variation along the ligament (5, 12). Additionally, the LOM contains the vein of Marshall, which is electrically connected to the CS through its muscular sleeve and to the LA free wall through Marshall bundles (1, 3–10). LOM activation patterns differ between patients, which is caused by differences in LOM anatomy and the number of connections between the LOM and the surrounding tissue, as demonstrated by Han et al. (15). In their study, patients were categorized into having single, double or multiple LOM connections, which was determined by application of differential pacing from the CS, left PVs, and LA appendage. In patients with a single connection, the LOM is only electrically connected to the CS, causing a proximal-to-distal activation sequence. Because the remainder of the LA is pre-excited by the sinus wavefront from Bachmann’s bundle, typical double potentials are visible during SR. This was also observed in the present study. In patients with double or multiple connections, the LOM is not only electrically connected to the CS, but also to the LA or PVs. During SR, wavefronts from Bachmann’s bundle and the CS are therefore competing and LOM potentials might not be clearly separable. This explains why LOM potentials can be difficult to distinguish from the LA potentials during SR in patients with double or multiple LOM connections.

### Role of the ligament of Marshall in arrhythmogenesis

The LOM may have different roles in the pathophysiology of atrial tachyarrhythmias. Firstly, focal activity may be present in the LOM and it can therefore serve as a trigger for atrial tachyarrhythmias (13, 14). Secondly, the LOM is influenced by the autonomic nervous system through its rich sympathetic and parasympathetic innervation, stimulation of which can induce arrhythmias. Thirdly, the LOM can serve as a bypass tract between the coronary sinus and pulmonary veins, when more than one connection is present, possibly facilitating macro-re-entry circuits (13, 15). The LOM can cause AF recurrences after PV isolation, through LOM-mediated PV reconnection (16). Also, the LOM can serve as a portion of a macro-re-entry circuit, for example in peri-mitral atrial tachycardias (17, 18). Additionally, the LOM causes localized areas of conduction block, which may contribute to the initiation and perpetuation of AF and thereby serve as a substrate for AF (35).

### Therapeutic options

Several recently published studies demonstrated a variety of therapeutic options targeting the LOM. This includes using a catheter-based, surgical or hybrid approach for additional endocardial or epicardial ablative lesions on the LOM in adjunct to PV isolation (13, 20, 24, 25). Hence, in these procedures, a linear catheter is preferred over a balloon-based catheter for endovascular PV isolation. Retrograde infusion of ethanol into the VOM after performing a CS venogram is another technique to ablate the LOM and has recently been investigated in several RCTs (21–23). This procedure has proven to be effective during a follow-up of 12 months in AF patients with arrhythmogenic activity in the LOM and decreases AF recurrence rates after combined catheter ablation and ethanol infusion. However, a potential disadvantage of VOM ethanol-infusion could be the lack of specificity in the area that is ablated, causing unnecessary damage of atrial tissue in an area larger than what is contributing to the arrhythmia.

### Clinical implications

Prior mapping studies of the LOM have mainly used bipolar LAT mapping strategies to identify the LOM and target it for treatment. However, we have demonstrated, by using a high-density epicardial mapping approach, that the LOM can be localized more accurately using unipolar EGMs in which both primary deflections of single potentials and secondary deflections of double and fractionated potentials are visualized in activation as well as voltage maps. In daily clinical practice, this signal processing approach may aid in visualizing the LOM real-time during both mapping guided arrhythmia surgery and endovascular interventions targeting the LOM. Real-time LOM visualization could lead to more specific ablation of solely the LOM, eliminating the need to ablate the entire area using VOM ethanol infusion, thereby bypassing its potential disadvantages.

### Limitations

Due to the strict nature of the selection criteria used to select LA locations containing a LOM, it is possible that the number of patients in which a LOM is found in this study is an underestimation. However, it was not the primary aim of this study to correctly identify the number of patients in whom a LOM could be detected.

Additionally, it is not possible to directly correlate the exact anatomical Marshall bundle structure and histological properties of the area mapped to the electrophysiological properties, which is inherent to the applied mapping technique. In a future prospective study, this could be partially addressed by confirmation by the surgeon that the electrode array is positioned on the LOM, which is visible and accessible after a small rightward shift of the heart. Besides, programmed electrical stimulation at the LOM could confirm the electrophysiological properties as demonstrated in the current study.

## Conclusion

Unipolar mapping of the LA with annotation and visualization of both primary deflections of single potentials and secondary deflections of double and fractionated potentials in activation and voltage maps is the most accurate signal recording and processing approach to electrophysiologically visualize the LOM. Additional studies are required to further develop this technique. The next step is intra-operative real-time visualization of the LOM using the signal recording and processing approach as discussed in this article, and to use this approach to guide ablation therapy targeting the LOM in patients undergoing arrhythmia surgery.

## Data availability statement

The datasets presented in this article are not readily available because of EU privacy law. Requests to access the datasets should be directed to corresponding author.

## Ethics statement

The studies involving human participants were reviewed and approved by METC Erasmus MC. The patients/participants provided their written informed consent to participate in this study.

## Author contributions

SL contributed to data acquisition and analysis, manuscript drafting, and conceptual thinking. MS contributed to data acquisition and analysis and critically revising the manuscript. AB and YT contributed to data acquisition and critically revising the manuscript. NG contributed to manuscript drafting and conceptual thinking. All authors contributed to the article and approved the submitted version.
